# Interaction of Src and Alpha-V Integrin Regulates Fibroblast Migration and Modulates Lung Fibrosis in A Preclinical Model of Lung Fibrosis

**DOI:** 10.1038/srep46357

**Published:** 2017-04-11

**Authors:** Yin-Ying Lu, Xue-Ke Zhao, Lei Yu, Fei Qi, Bing Zhai, Chang-Qing Gao, Qiang Ding

**Affiliations:** 1 Comprehensive Liver Cancer Center, 302 hospital, Beijing, China; 2 Department of Infectious Diseases, the Hospital Affiliated to Guizhou Medical University, Guiyang, Guizhou, China; 3 Department of Medicine, Pulmonary Division, University of Alabama at Birmingham, Alabama, USA; 4 Prenatal Diagnostic Center, The Affiliated Hospital of Guizhou Medical University, Guiyang 550004, Guizhou, China; 5 Department of Respiratory Medicine, PLA General Hospital, Beijing, China; 6 Department of Geriatric Hematology, PLA General Hospital, Beijing, China; 7 Research Center for Medical Sciences, The Third Xiang-Ya Hospital and the Department of Laboratory Animals, Xiang-Ya School of Medicine, Central South University, Hunan, China

## Abstract

Src kinase is known to regulate fibroblast migration. However, the contribution of integrin and Src kinase interaction to lung fibrosis has not been mechanistically investigated. Our data demonstrate that integrin alpha v (αV) recruited Src kinase and that leads to subsequent Src activation in fibroblasts plated on fibrotic matrix, osteopontin. Src interaction with integrin αV is required for integrin αV-mediated Src activation, and the subsequent fibroblast migration. The study identified that β5 and β3 are the major integrins for this effect on osteopontin. In contrast, integrins β1, β6, and β8 did not have a critical role in this phenomenon. Importantly, Src inhibitor significantly reduces fibroblast migration stimulated by PDGF-BB and reduced *in vivo* lung fibrosis in mice. Src inhibitor reduced Src activation and blocked the signaling transduction by integrin αV, inhibited migration signaling pathways and reduced extracellular matrix protein production, and blocked myofibroblast differentiation *in vivo* in mouse lung tissues. The present study supports that the interaction of Src Kinase and integrins plays a critical role in the development of lung fibrosis and the signaling involved may present a novel opportunity to target deadly fibrotic diseases.

Pulmonary fibrosis (PF) is a chronic lung disease that results in a progressive decline in lung function and death with a mean survival of about 5 years^1^,^2^. While the pathogenesis of this disease is not fully understood, there does appear to be alterations in fibroblast migration and excessive matrix deposition^3^,^4^,^5^,^6^,^7^,^8^,^9^. Fibroblast migration into wounded areas and differentiate into myofibroblasts are known as the critical contributing factors leading to either normal wound healing and excessive tissue remodeling, fibrotic diseases including PF. Persistent fibroblast migration contributes to the expansion of fibrotic lesions in multiple organs and results in excessive tissue remodeling^10^. During fibrotic remodeling, many cytokine and growth factors are expressed and released; some are potent mediator of cell migration, for example, PDGF-BB (platelet-derived growth factor) is expressed and released during fibrotic remodeling. PDGF-BB has been implicated in many fibrotic diseases, including lung fibrosis^11^, liver fibrosis^8^, atherosclerosis^9^, and skin fibrosis^12^. PDGF-BB is well known for its function in promoting cell migration, particularly in cancer cell migration and fibroblast cell migration^13^,^14^.

Fibroblasts isolated from human fibrotic organs show increased ability to migration and seem to obtain a migratory phenotype^3^,^4^. Fibroblast migration and proliferation result in the development of the leading edges of progressive fibrotic lesions and the formation of fibrotic lesions generally are able to penetrate the surrounding tissues and cause lung epithelial death and lung tissue destruction. Fibroblast migration and proliferation are tightly controlled and regulated by the integrin receptors and the interaction between integrin receptors and extracellular matrix^3^,^14^.

Cell migration is a complex and coordinated biological process. Protein kinase regulated cell migration is involved in the development of lung fibrosis^4^. Src kinase regulates focal adhesion kinase activation. Src kinase binds to focal adhesion kinase and activates it through phosphorylation of the tyrosine 397 (Y397)^15^,^16^,^17^. The Src family kinases are a group of non-receptor tyrosine kinases, and regulate broad cell functions, including migration, invasion, and growth^18^,^19^,^20^,^21^,^22^,^23^,^24^. Src kinase is activated by autophosphorylation of tyrosine residue 418 (Y418)^18^,^21^. The c-terminal domain of Src kinase is often myristoylated or palmitoylated to allow for association with the cell membrane receptors, such as integrins^18^,^25^. This association facilitates the binding of Src kinase to other signaling proteins around focal adhesions or integrins, and activate them^18^,^21^,^23^. Although it is known that integrins and Src activation are involved in myofibroblast differentiation and lung fibrosis^26^, the role of integrin and Src kinase interaction in fibroblast migration and proliferation, and in lung fibrosis *in vivo*, is still underexplored.

In this study, we show that pro-fibrotic factor, platelet derived growth factor BB (PDGF-BB), induces Src activation and lung fibroblast migration. We provide evidences that Src is directly associated with integrin alpha-V (αV) in PDGF-BB stimulated lung fibroblasts. The association of Src and integrin αV is important for PDGF-BB stimulated fibroblast migration on osteopontin. Our data demonstrate that integrin αV recruited Src kinase and that leads to subsequent Src activation in fibroblasts plated on fibrotic matrix, osteopontin. The study identified that β5 and β3 are the major integrins for this effect on osteopontin. Importantly, Src inhibitor significantly reduces fibroblast migration stimulated by PDGF-BB and reduced *in vivo* lung fibrosis in mice. The present study supports that the interaction of Src Kinase and integrins plays a critical role in the development of lung fibrosis and the signaling involved may present a novel opportunity to target deadly fibrotic diseases.

## Results

### Src is recruited and directly associated with integrin αV in response to PDGF-BB treatment in fibroblasts

**Figure 1:**
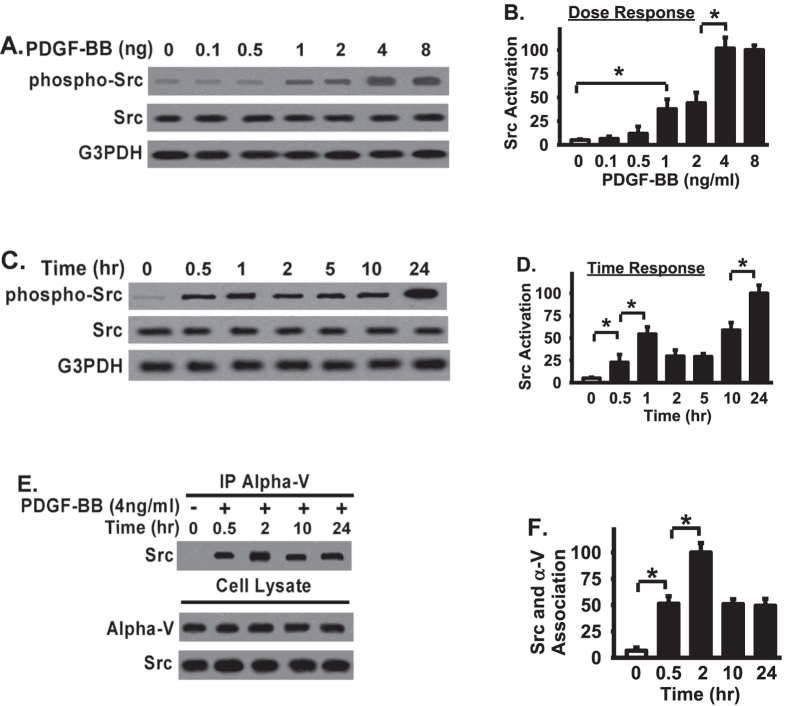
Src is activated in a dose-dependent and time-dependent manner, and directly associated with integrin αV in response to PDGF-BB treatment in fibroblasts. (**A**) Fibroblasts were planted on osteopontin (10 μg/mg), serum starved, treated with PDGF-BB for overnight, and lysed for Western blot. (**B**) Densitometry of Src activation. pY416 of Src levels were normalized to total Src levels. Data were represented as the percentage of Src activation relative to that in highest dose (8 ng/ml, set as 100%). (**C**) Cell lysates were Western blotted with indicated antibodies. (**D**) Densitometry of Src activation from Panel C. pY416 of Src levels were normalized to total Src levels. Data were represented as the percentage of Src activation relative to that in longest time point (24 hours, set as 100. (**E**) Whole cell lysates were used for coimmunoprecipitation with anti- αV IgG, followed by Western blotted with anti-Src antibody to examine the Src-αV interaction. (**F**) Densitometry of Src-αV association from Panel E. Data were represented as the percentage of association elative to that in longest time point (24 hours, set as 100%). *Represents < 0.01.

Osteopontin is excessively produced and abundantly exists in fibrotic lesions, and reduced osteopontin expression is associated with reduced tubulointerstitial fibrosis^27^. However, little information is currently available regarding the specific integrin receptor(s) involved in fibrotic diseases. As Src senses the extracellular stimuli and initiates signaling cascades which are important for cells to correctly response to the extracellular stimuli under different situations^14^, we first examine the interaction between Src and osteopontin receptor integrin αV in response to one important pro-fibrotic factor, platelet-derived growth factor BB (PDGF-BB), in fibroblasts. Human lung fibroblasts were serum starved, plated into osteopontin-coated (10 ug/ml) tissue culture wells, and subjected to Western blot assays for Src activation. Src activation was evaluated through tyrosine 416 phosphorylatio of Src (phosphor-Src), a prominent phosphorylation site of Src kinase^18^. Src activation induced by PDGF-BB is dose-dependent and time-dependent. PDGF-BB-induced Src activation reached two peaks, one is at the range of 1–2 ng/ml and the other peak is at the range of 4–8 ng/ml (Fig. 1A,B). In contrast, PDGF-BB did not induce significant Src activation in fibroblasts when PDGF-BB was less than 0.5 ng/ml (Fig. 1A,B). Src was activated as early as 30 minutes after PDGF-BB stimulation at 4 ng/ml dose in human lung fibroblasts (Fig. 1C,D). A late but strong Src activation was shown 24 hrs after PDGF-BB stimulation (Fig. 1C,D). Src activation is steadily increased from 0.5–10 hours after PDGF-BB treatment and reached a peak at 24 hours after PDGF-BB treatment (Fig. 1C,D).

Although it is known that Src can be activated by interaction with integrins^19^,^25^,^28^,^29^,^30^, the specific role of osteopontin receptor, integrin αV, on Src activation in the context of pro-fibrotic response has not been particularly investigated. We investigated the interaction among Src and integrin αV by using coimmunoprecipitation assays (Fig. 1E). Human lung fibroblasts were serum starved, treated with PDGF-BB (4 ng/ml), and whole cell lysates were immunoprecipitated with specific anti-Src antibody at indicated time points (Fig. 1E). The findings indicate that Src was not associated with integrin αV without PDGF-BB stimulation in fibroblasts (Fig. 1E,F). Upon PDGF-BB stimulation, Src was directly associated with integrin αV and the association was peaked 2 hours after PDGF-BB stimulation (Fig. 1E,F). These data demonstrate that Src is activated by PDGF-BB in a dose-dependent and time-dependent manner, and Src is recruited and directly associated with integrin αV in response to PDGF-BB treatment in fibroblasts.

### PDGF-BB-induced fibroblast migration is dose-dependent and time-dependent on osteopontin; Inhibition of Src activation decreases PDGF-BB-induced fibroblast migration but does not block the Src association with osteopontin receptor integrin αV

**Figure 2:**
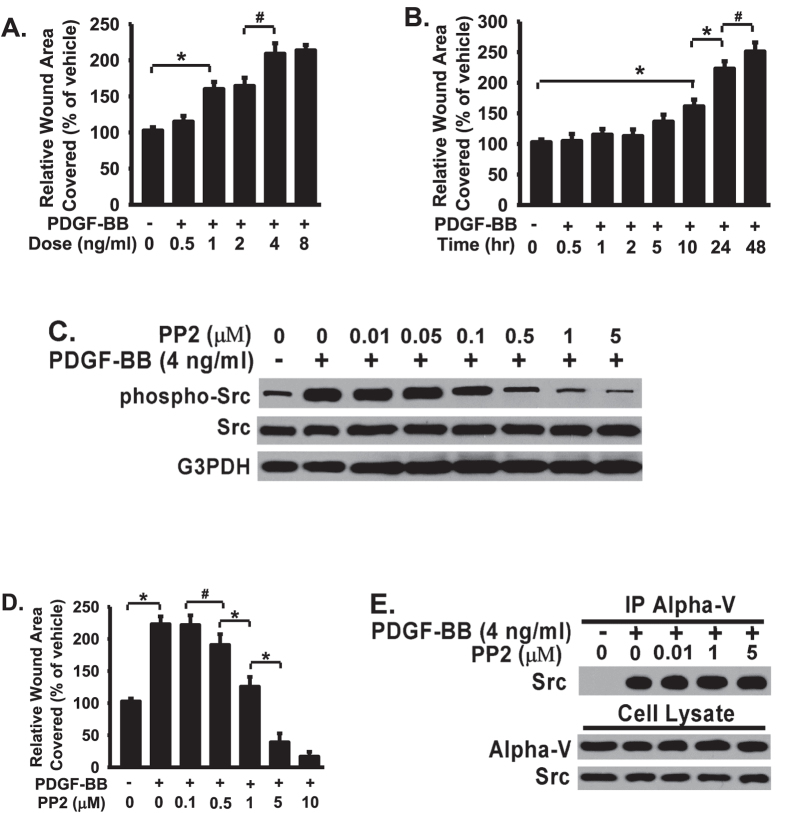
PDGF-BB-induced fibroblast migration is dose-dependent and time-dependent on osteopontin; Inhibition of Src activation decreases PDGF-BB-induced fibroblast migration but does not block the Src association with osteopontin receptor integrin αV. (**A**) Fibroblasts were plated on osteopontin (10 μg/mg), serum-starved, wounded, treated with PDGF-BB or vehicle, and wound area was monitored for 24 hours at 37 °C. Data plotted as % of wound area covered over 24 hours relative to vehicle treated fibroblasts. (**B**) Fibroblasts were treated as Panel A and with PDGF-BB (4 ng/ml) or vehicle. (**C**) Fibroblasts were treated as Panel A with PDGF-BB (4 ng/ml), followed by Src inhibitor PP2 with indicated dose overnight, and lysed Western blotted. (**D**) Fibroblasts were wounded and treated with PDGF-BB (4 ng/ml) or vehicle, followed by PP2 (1 μM), and the monolayer wound area was monitored for 24 hours at 37 °C. (**E**) Fibroblasts were treated as in Panel A, followed by PDGF-BB (4 ng/ml) and Src inhibitor PP2 overnight. Whole cell lysates were used for coimmunoprecipitation with anti-αV, followed by Western blotted with anti-Src antibody to examine the Src-αV interaction. *Represents < 0.01. #Represents < 0.05.

Src is well known for its role in promoting cell migration^14^,^18^,^25^,^31^,^32^,^33^. The above data show that Src is recruited and directly associated with integrin αV in response to PDGF-BB treatment in fibroblasts plated on oteopontin (Fig. 1E,F). We next investigated the specific role of Src association with integrin αV on PDGF-BB stimulated fibroblast migration. First, fibroblast migration on osteopontin in response to PDGG-BB stimulation has been studied by the wound closure assay at the indicated dose- and time (Fig. 2). Control and PDGF-BB-stimulated fibroblasts migration was determined and measured. The relative wound areas covered (or the areas invaded by migrating fibroblasts) within 24 hours were quantified by densitometry and the findings were analyzed (Fig. 2). PDGF-BB induced fibroblast migration in a dose-dependent manner within 24 hours (Fig. 2A). PDGF-BB-stimulated migration rate (wound covered area per 24 hour) is positively associated the dose used, with highest migration rate at the range of 4–8 ng/ml (Fig. 2A). PDGF-BB induced fibroblast migration is also in a time-dependent manner. The migration rate was significantly increased at 10 hours and last up to 48 hours respectively after PDGF-stimulation (Fig. 2B). The maximal fibroblast migration rate (Fig. 2A,B) is corresponding to maximal Src activation (Fig. 1C,D) in fibroblasts.

To further understand the role of Src-integrin αV interaction in PDGF-BB-induced cell migration, fibroblasts were treated with or without Src inhibitor PP2 at indicated concentrations followed by monolayer wound healing closure assay. PP2 inhibits PDGF-BB-induced Src activation (pY416 of Src, phosphor-Src) and PDGF-BB-induced fibroblast migration in a dose-dependent manner (Fig. 2C,D, respectively). PP2 inhibition of fibroblast migration (Fig. 2D) is closely associated with its ability to inhibit Src activation (Fig. 2C), supporting that Src activation is required for PDGF-BB-induced fibroblast migration. Interestingly, Src inhibitor treatment did not block the association between Src and integrin αV, evidenced by that PP2 had no effect on Src- integrin αV interaction (Fig. 2E), even at the dose (1 or 5 μM) which effectively inhibited PDGF-BB-induced Src activation and fibroblast migration (Fig. 2D,E). The above data demonstrate that Src activation in response to PDGF-BB is both dose- and time-dependent, and Src activation is absolutely required for PDGF-BB-induced fibroblast migration. The data also suggest that Src-integrin αV interaction may be upstream of Src activation, as Src inhibitor blocks Src activation and fibroblast migration on osteopontin but has no effect on Src-integrin αV interaction.

### Integrins αVβ5 and αVβ3 are main integrin receptors contributing to Src-mediated fibroblast migration and Src activation on osteopontin

**Figure 3:**
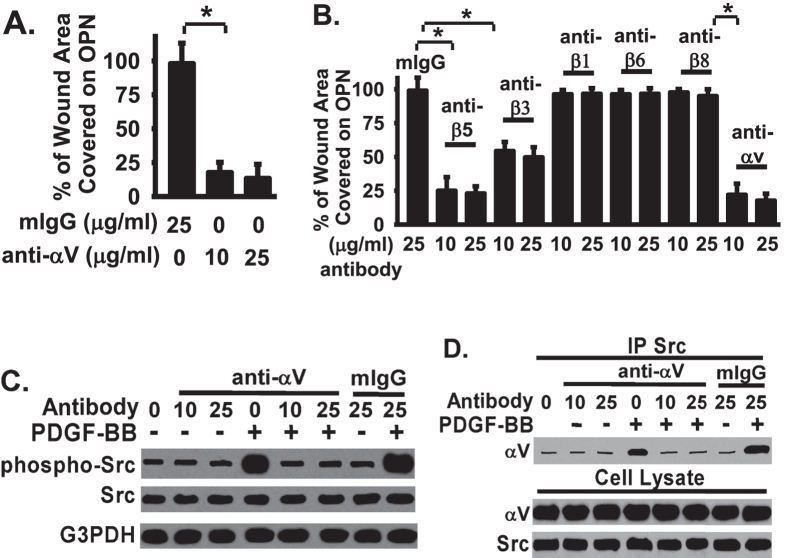
Integrins αVβ5 and αVβ3 are main integrin receptors contributing to Src-mediated fibroblast migration and Src activation on osteopontin. (**A**) Fibroblasts were planted on osteopontin (OPN, 10 μg/mg), serum starved, wounded as in Panel A, treated with PDGF-BB (4 ng/ml), followed by αV integrin blocking antibody. The monolayer wound area was monitored for 24 hours at 37 °C. Data plotted as % of wound area covered over 24 hours relative to control IgG treated fibroblasts (bar 1, defined as 100%). (**B**) Fibroblasts were treated as in Panel A and with PDGF-BB (4 ng/ml), followed by indicated integrin blocking antibodies or control mouse IgG. The monolayer wound area was monitored for 24 hours at 37 °C. Data were plotted as % of wound area covered over 24 hours relative to control IgG treated fibroblasts. (**C**) Fibroblasts were treated as in Panel A and lysed for Src activation. (**D**) Fibroblasts were treated as in Panel A and lysed. Whole cell lysates were used for coimmunoprecipitation with anti-αV IgG, followed by Western blotted with anti-Src antibody to examine the Src-αV interaction. *Represents < 0.01.

The above data (Fig. 2) suggest that Src-integrin αV interaction may be upstream of Src activation, as Src inhibitor blocks Src activation and fibroblast migration on osteopontin but has no effect on Src-integrin αV interaction. To examine whether integrin αV interacts with osteopontin and whether this is upstream of Src activation, we first used blocking antibody against integrin αV and investigated its effect on fibroblast migration on osteopontin. Fibroblasts were plated into osteopontin-coated tissue culture wells, serum starved, treated with integrin αV blocking antibody or control mouse IgG (mIgG) at indicated dose, and migration stimulated by PDGF-BB (4 ng/ml) was determined by monolayer wound healing closure assay. Osteopontin has multiple integrin receptors, including αVβ5, αVβ3. Integrin αV blocking antibody significantly blocked PDGF-BB-stimulated migration on osteopontin (Fig. 3A). Integrin αV blocking antibody reduced about 91% migration when compared to control IgG, p < 0.001), demonstrating that Integrin αV is the main integrin osteopontin receptor mediating the PDGF-BB-stimulated fibroblast migration on osteopontin. Integrin β5 or β3 blocking antibody inhibited about 75% or 51% of fibroblast migration on osteopontin (Fig. 3B), suggesting that αVβ5 and αVβ3 have overlapping functions in mediating PDGF-BB-stimulated migration on osteopontin. Blocking integrin β1, β6, or β8 individually inhibited about 6–8% of fibroblast migration on osteopontin (Fig. 3B), indicating that they play a minor role. This is further supported by that blocking integrin αv inhibited about 92% of fibroblast migration on osteopontin (Fig. 3B), supporting that integrin β1, β6, and β8 play a minor role in mediating fibroblast migration on osteopontin.

As Integrin αV is the main integrin osteopontin receptor mediating the PDGF-BB-stimulated fibroblast migration on osteopontin (Fig. 3A), we next studied the role of integrin αV binding to osteopontin on Src activation. Integrin αV blocking antibody significantly blocked PDGF-BB-stimulated Src activation (Fig. 3C). Integrin αV blocking antibody or control IgG did not block unstimulated Src activation in cells plated on osteopontin (Fig. 3C, without PDGF-BB). Importantly, integrin αV blocking antibody interrupted the association of Src with integrin αV in PDGF-BB-stimulated fibroblasts (Fig. 3D), demonstrating that integrin αV binding to osteopontin is necessary for Src association with integrin αV and subsequent Src activation.

### Src inhibitor attenuates lung fibrosis in a preclinical mouse lung fibrosis model, in bleomycin-challenged mice

**Figure 4:**
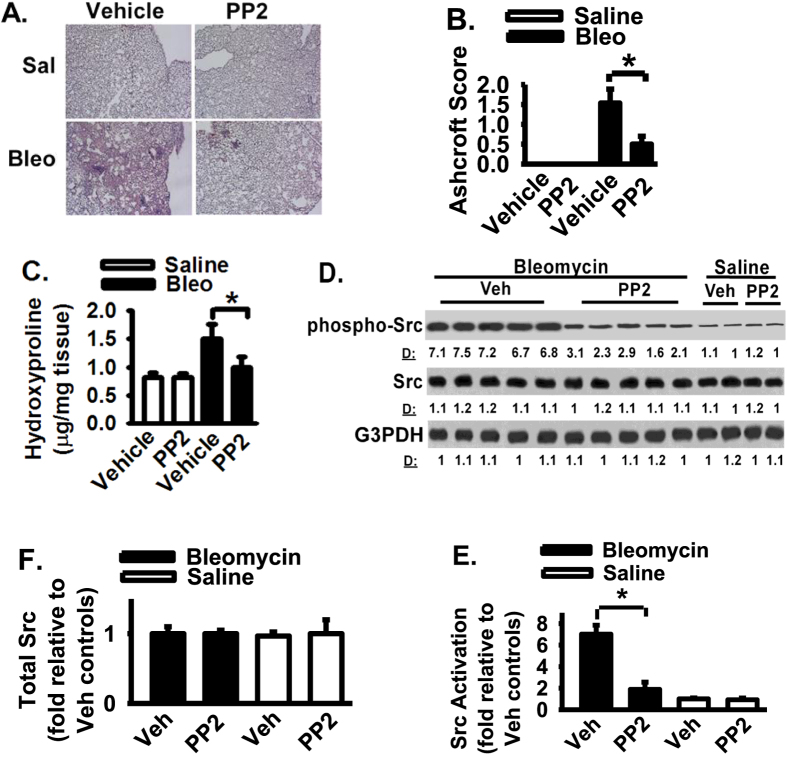
Src inhibitor protects lung fibrosis in bleomycin-challenged mice. (**A**) Mice were instilled with saline (Sal) or bleomycin (Bleo) and followed by daily treatment of PP2 (50 mg/kg, i.p. injection) or vehicle. Lung tissues at day 21 were HE stained (200×). (**B**) The severity of lung fibrosis was examined morphometrically and represented by Ashcroft Score. (**C**) Lung hydroxyproline level was measured and represented as % hydroxyproline normalized to that in vehicle-challenged mice. (**D**) Lung tissue lysates were Western blotted. (**E**) Densitometry of Src activation from Panel D. pY416 of Src levels were normalized to total Src levels. Data were represented as the fold of Src activation relative to that in the control vehicle and saline treated group. (**F**) Densitometry of total Src protein from Panel D. Src levels were normalized to G3PDH levels. Data were represented as the fold of Src relative to that in the control vehicle and saline treated group. Per experimental group had 8–12 mice. Data were represented as mean + SE. * represents p < 0.01.

To determine the functional role of Src in the development of lung fibrosis, the effect of Src inhibitor on lung fibrosis was determined by using the animal model of lung fibrosis induced by bleomycin. Bleomycin is widely used as a chemotherapy reagent but has a toxic side effect and induces lung fibrosis in human and mouse^34^. Animals were challenged with bleomcyin or saline, followed by treatment of PP2 or vehicle (saline) daily for 21 days. Src inhibitor PP2 protects lung fibrosis in bleomycin-challenged animals (Fig. 4A). Morphometric analysis of lung tissue sections reveals an approximately 1.3-fold decrease (p < 0.01) in fibrotic score in the bleomycin-challenged mice treated with Src inhibitor, when compared to that in bleomycin-challenged mice treated with vehicle only (Fig. 4B, Ashcroft score). There was no difference in the minimal fibrotic score observed in the lung parenchyma between animals treated with Src inhibitor or control vehicle in the unchallenged state (Fig. 4B). Using the quantifiable measure of hydroxyproline for the total collagen content in whole lung tissues, Src inhibitor significantly decreased total collagen levels in bleomycin-challenged mice when compared to that in vehicle treated mice (Fig. 4C, about a 1.25-fold decrease, p < 0.01), demonstrating the Src inhibitor significantly reduced lung fibrosis. No differences on basal collagen content were noted between animals treated with Src inhibitor and animals treated with vehicle only (Fig. 4C) or animals in unchallenged/basal conditions (data not shown). Src activation is significantly increased in response to bleomycin challenge when compared to vehicle saline treatment (Fig. 4D, lanes 1–5 versus lanes 11–12). Src inhibitor PP2 significantly reduced Src activation in bleomycin-treated lungs (Fig. 4D, lanes 6–10 versus lanes 1–5), supporting that Src inhibitor blocks Src activation and attenuates lung fibrosis in animal model of lung fibrosis.

### Src inhibitor decreases activation and expression of proteins that mediate cell migration and myofibroblast differentiation *in vivo*

**Figure 5:**
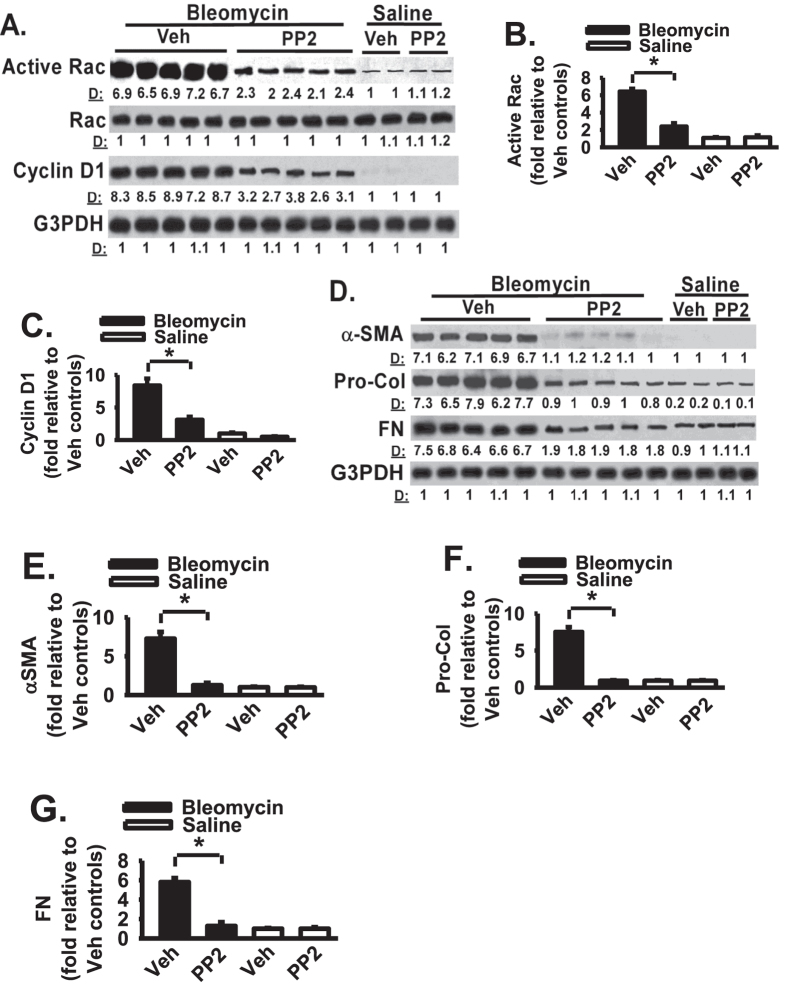
Src inhibitor decreases activation and expression of proteins that mediate cell migration, proliferation, and myofibroblast differentiation *in vivo*. (**A**) Mice were instilled with saline or bleomycin and followed by daily treatment of PP2 or vehicle (Veh). Lung tissue lysates at day 21 were Western blotted. (**B**) Relative quantification of active Rac in Panel A. (**C**) Relative quantification of cyclin D1 in Panel A. (**D**) Lung lysates were Western blotted with indicated antibodies. (**E**) Relative quantification of alpha-SMA in Panel C. (**F**) Relative quantification of pro-collagen in Panel C. (**E**) Relative quantification of fibronectin (FN) in Panel C.

It is likely that Src inhibitor protects lung fibrosis by blocking multiple pro-fibrotic pathways and mechanisms in bleomycin-challenged mice. As above data support that Src plays an essential role in fibroblast migration, one possible underlying mechanism for the protective effect of Src inhibitor is a decrease in mesenchymal cell or fibroblast migration or proliferation as Src is known to promote cancer cell proliferation^30^,^31^,^35^,^36^. We therefore assessed the effect of Src inhibitor on the activity/expression of key cell migration regulatory proteins *in vivo* (i.e., Src, Rac, and cyclin D1, a cell proliferation driver). We first noted that activation of Src (pY416 of Src, phosphor-Src) and Rac (GTP-bound form), and increased cyclin D1 expression, are part of the fibrogenic process, as their active form (Src and Rac) or expression of cyclin D1 is increased in bleomycin challenged mice when compared to non-fibrotic, saline-challenged controls (Figs 4D and 5A, respectively). Src inhibitor significantly decreased Src activation (Fig. 4D) and active Rac (Fig. 5A) in bleomycin-challenged mice when compared to vehicle treated mice. Src inhibitor significantly reduced cyclin D1 expression in bleomycin-challenged mice when compared to vehicle treated mice (Fig. 5A). Whole lung fibronectin and procollagen-1 levels after bleomycin challenge are increased in mouse lung tissues when compared to that in saline-treated control mice (Fig. 5C, lanes 1–5 versus lanes 11–12). Src inhibitor significantly decreased the whole lung fibronectin and procollagen-1 levels in bleomycin-challenged mice when compared to vehicle treated mice (Fig. 5C, lanes 1–5 versus lanes 6–10). In addition, Src inhibitor significantly reduced α-smooth muscle actin (SMA) expression, a marker for myofibroblast, in bleomycin-challenged mice when compared to vehicle treated mice (Fig. 5C, lanes 1–5 versus lanes 6–10). These data demonstrate that Src inhibitor blocks activation and expression of proteins that mediate cell migration and myofibroblast differentiation *in vivo*.

## Discussion

Our study demonstrates that PDGF-BB induces Src activation and lung fibroblast migration in a dose-dependent and time-dependent manner. Upon PDGF-BB stimulation, Src is quickly and directly associated with integrin αV in PDGF-BB stimulated lung fibroblasts plated on osteopontin. The association of Src and integrin αV is essential for Src activation and Src-mediated fibroblast migration. Our results first time show that Src mediates PDGF-BB stimulated fibroblast migration largely via integrin. Integrin αVβ5 and αVβ3 contribute to about 90% of this effect and their function is overlapped. Integrins β1, β6, and β8 contribute to about 5% effects of Src-mediated fibroblast migration on osteopontin. Src inhibitor significantly reduces PDGF-BB stimulated fibroblast migration. Importantly, our data demonstrate that Src inhibitor protects bleomycin-induced lung fibrosis in mice. These data demonstrate that Src mediates fibroblast migration mainly via integrin integrin αV and targeting Src signaling is an effective therapeutic strategy against fibrosis.

Persistent development and expansion of fibrotic lesions leads to persistent fibrotic remodeling in affected organs. PDGF-BB is a pro-fibrotic factor. PDGF-BB has been implicated in many fibrotic diseases, including lung fibrosis^11^, liver fibrosis^8^, atherosclerosis^9^, and skin fibrosis^12^. PDGF-BB is well known for its function in promoting cell migration, particularly in cancer cell migration and fibroblast cell migration^13^,^14^. The PDGF-BB level is increased in fibrotic response^8^,^9^,^11^,^12^; therefore, the formation of these “leading edges” in fibrotic tissues likely results from persistent fibroblast migration stimulated by PDGF-BB). Fibrotic fibroblasts from human fibrotic lung tissues show increased cell migration^3^. That likely contributes to development of the leading edges of progressive fibrotic lesions, as well as facilitates the formation of fibrotic reticulum penetrating the surrounding tissues and extends the fibrotic scar into surrounding tissues. Cell migration is a complex and coordinated biological process. It is likely that the combination of cell signaling initiated by integrins and ECM may direct fibroblast migration and the direction of fibroblast migration^3^,^14^; however, their functions in fibrosis have not been completed revealed. TGF-β1 is known as a major fibrotic mediator by increasing ECM protein accumulation, and it regulates lung fibrosis through signaling pathways mediated by Src or other integrin associated proteins^4^,^37^. However, TGF-β1 is less potent to induce cell migration but stimulates myofibroblast phenotype and epithelial to mesenchymal transition^38^,^39^. PDGF-BB has been shown to increase cell migration in primary lung cells isolated from pulmonary fibrosis patients^40^.

Src is required for the signaling cascade initiated by the interaction between integrins and ECM proteins, such as osteopontin. Our data demonstrate that Src activation is dependent upon the PDGF-BB dose, as well as the treated time (Fig. 1A,D). The data indicate that Src is quickly recruited and directly associated with integrin αV in response to PDGF-BB treatment in fibroblasts plated on osteopontin (Fig. 1E,F). Our data support that osteopontin likely contributes to persistent fibrotic development through Src activation and Src-mediated fibroblast migration. However, little information is currently available regarding the specific integrin receptor(s) involved. The findings demonstrate that integrins αVβ3 and αVβ5 are main integrin receptors contributing to Src-mediated fibroblast migration and Src activation on osteopontin (Fig. 3). In contrast, integrin β1, β6, or β8 play a minor role in mediating fibroblast migration on osteopontin (Fig. 3), but they may have distinct role in regulating cellular function which are not specifically investigated in the present study. Integrin integrin αV plays a major role in Src activation induced by PDGF-BB (Fig. 3). Integrin integrin αV blocking antibody largely blocked PDGF-BB-stimulated Src activation (Fig. 3), as well as interrupted the association of Src with integrin integrin αV in PDGF-BB-stimulated fibroblasts (Fig. 3). These data demonstrate that integrin integrin αV binding to osteopontin is necessary for Src association with integrin integrin αV and subsequent Src activation in response to PDGF-BB.

Src inhibitor PP2 inhibits PDGF-BB-induced Src activation and fibroblast migration in a dose- and time-dependent manner (Fig. 1). Interestingly, Src inhibitor treatment did not block the association between Src and integrin integrin αV, evidenced by that PP2 had no effect on Src- integrin αV interaction (Fig. 1E), even at the dose (1 or 5 μM) which effectively inhibited PDGF-BB-induced Src activation and fibroblast migration (Fig. 1D,E). These data demonstrate that Src activation is absolutely required for PDGF-BB-induced fibroblast migration, which has not known previously. At the same time, the above data also demonstrate that Src activation requires the integrin αV-binding to osteopontin and Src-integrin αV interaction is upstream of Src activation.

As Src senses the extracellular stimuli and initiates signaling cascades which are important for cells to correctly response to the extracellular stimuli under different situations, targeting Src signaling may interrupt the persistent fibrosis. To determine the functional role of Src in the development of lung fibrosis, the effect of Src inhibitor on lung fibrosis was determined by using the animal model of lung fibrosis induced by bleomycin. Bleomycin lung fibrosis model is controversial but is widely used and extensively published^34^,^41^,^42^,^43^. Src inhibitor protects lung fibrosis in bleomycin-challenged animals (Fig. 4). Src inhibitor significantly inhibited Src activation in bleomycin-challenged mice. Src inhibitor significantly reduced fibrotic score and total lung collagen content in bleomycin-challenged mice, supporting that Src inhibitor is protective against the pro-fibrotic effects of bleomycin. These data supporting that Src inhibitor blocks Src activation and attenuates lung fibrosis in animal model of lung fibrosis. Our findings are consistent with previous published data that inhibition of Src kinase has been shown to inhibit hypertrophic scar and lung fibrosis^26^,^44^. Our results confirm the anti-fibrotic effects published previously and extend to further understand the underling molecular mechanisms. It is likely that Src inhibitor protects lung fibrosis by blocking multiple pro-fibrotic pathways and mechanisms in bleomycin-challenged mice. Our findings support that one underlying mechanism for the protective effect of Src inhibitor is a decrease in fibroblast migration and proliferation in animals treated with Src inhibitor. Src inhibitor significantly reduced the activity/expression of key cell migration and proliferation regulatory proteins (i.e., Src, Rac, and cyclin D1) in bleomycin-challenged mice. During cell migration, cytoskeletal actin polymerization often lead to lamellipodia formation mediated by GTPase, Rac^45^,^46^,^47^. Rac mediated cell signaling is important for cell migration^45^,^46^,^47^. Rac has been implicated in cell migration downstream signaling of Src^45^,^46^,^47^. In fact, fibrotic lung tissues (from bleomycin-challenged mice) show significant activation of Src and Rac (GTP-bound form), and significant increased cyclin D1 expression; therefore, they are part of the fibrogenic process (Figs 4 and 5). These data support that Src inhibitor decreases activation and expression of proteins that mediate cell migration and proliferation, and myofibroblast differentiation. Additional supports are that Src inhibitor decreased the whole lung fibronectin and procollagen-1 levels, and reduced α-SMA expression (a myofibroblast differentiation marker) in bleomycin-challenged mice. Myofibroblast differentiation is one main driving force of fibrosis^48^,^49^. These data suggest that Src inhibitor blocks the development of fibrotic lesions at least through inhibition of fibroblast migration, ECM protein production, and myofibroblast differentiation.

Increased cell migration during fibrotic reactions is continuously supported^3^,^50^,^51^,^52^,^53^. Understanding the signaling pathways contributing to increased fibroblast migration is important to develop more effective therapy for lung, liver, and other fibrosis as protein kinase and GTPase can be targeted and are involved^14^,^54^,^55^,^56^. This current study is limited to know whether manipulation of cell migration signaling downstream of Src will change the cellular behavior and lung injury and repair in response to pro-fobrotic stimuli. We demonstrate that increased activation of Src and Rac, as well as increased cyclin D1 expression in fibrotic lung tissues, and Src inhibitor significantly inhibited these effects. Fibroblast migration has been considered as one important factor of fibrosis, a dysregulated Src signaling may promote fibroblast migration and that could be one mechanism for the progressive fibrosis in lung, liver, cardiovascular system, kidney, and other organs. Pharmacological reagents targeting the dysregulated Src signaling^57^ or Src interaction with ECM protein via specific integrins may have potential effective and therapeutic effects for fibrosis in these vital organs.

## Methods

### Reagents

The following reagents and antibodies were commercially purchased: PDGF-BB (R&D Systems), anti-phospho-Src [pY416] (Cell Signaling), anti-Src (Upstate Biotechnology), mouse IgG (Molecular Probes), beta-1 (β1), β6, αVβ3, αVβ5, αV, and β8 (Abcam), pro-collagen, fibronectin (FN), anti-cyclin D1, and beta-actin (Santa Cruz Biotechnology), PP2, anti-α-SMA mouse IgG, fibronectin (FN), bleomycin, and other chemicals (Sigma).

### Cell Culture and Western Blotting

Normal human lung fibroblasts were from the American Type Culture Collection (ATCC) (Manassas, VA). Fibroblasts were cultured in Dulbecco’s modified Eagle’s medium containing 10% FBS and supplemented with penicillin/streptomycin/gentamycin. Early passages were used. Fibroblasts were lysed in detergent buffer (with 1% NP-40 and inhibitors: PMSF, Aprotinin, Leupeptin, Sodium Vanadate, and TLCK). The BCA kit (Pierce, Rockford, IL) was used to determine the protein concentration of cell or lung lysate. Same amount of whole cell detergent or lung tissue lysates were Western blotted by 12% SDS PAGE gel and transferred to Immobilon membrane, incubated with indicated antibodies, and developed with ECL system (Pharmacia). For loading control, the expression of beta-actin was used. Densitometric analysis of band intensity was determined by a pool of films by using standard analysis software (Adobe Photoshop).

### Wound closure monolayer/scratch motility assay

Cells were harvested with buffered EDTA, suspended in DMEM with 1% BSA, and plated into tissue culture plates (average 1 × 10^5^ cells/well in 24 well format). The cell migration and haptotactic fibroblast migration assays were performed at 24 hour. The cells was scratched, digital pictures were obtained immediately after scratching and again at the end of the assay. To calculate the areas of wound without cells after scratching and the remaining areas without cells at the end, the digital images were used. The covered wound area due to cell migration was equal to the difference between the begin and end areas, and the results were normalized to the control-treated cell migration groups. The osteopontin-mediated cell migration was examined in Boyden-type chambers (8 μm), cells (4 × 10^4^ cells) were plated onto osteopontin (10 μg/ml) coated bottom surface in DMEM with 1% BSA, and the cell migration was studied for 6 hours at 37 °C and 5% CO_2_. Cells were removed from the upper surface of the filter. The migrated cells on the lower filter surface were fixed, stained, and counted. Data are presented as the mean ± SE.

### Coimmunoprecipitation Assays

*Coimmunoprecipitation* analysis assays was performed. Briefly, equivalent microgram of protein lysate from each sample was incubated with sepharose-coupled antibody toward Src or Alpha-V overnight at 4 degree, washed, subjected to 12% SDS–PAGE gel Westeren blot analysis.

### *In Vivo* Lung Fibrosis Animal Model and Lung Fibrosis Analysis

All animal interventions were approved by local IACUC at Guizhou Medical University, Central South University, and University of Alabama at Birmingham. All methods were performed in accordance with the relevant ethical guidelines and regulations. Mice (C57Bl6, 8–11 weeks, female and male) were anesthetized and bleomycin (2 U/kg body weight in 50 μl saline) or saline vehicle alone (50 μl) was slowly instilled through airway to lungs by using an intratracheal catheter. For animals treated with Src inhibitor, the animals were i.p injected everyday with 50 mg/kg PP2 based on information provided by vendor and published data. Control animals were i.p injected everyday with vehicle. The animals were euthanized and lung tissues were harvested at day 21 after bleomycin treatment. The lung tissues were used for histological and biochemical studies, and for protein and cell migration signaling analysis. To collect lung tissue for histological studies, the lung tissues were fixed in paraffin. To collect lung tissue for histological studies, the lung tissues were fixed and paraffin embedded. Analysis of whole lung protein extracts was performed. Whole lung homogenates were prepared in detergent buffer (1% NP-40) with inhibitors (100 μM phenylmethanesulfonyl fluoride (PMSF), 10 μg/ml Aprotinin, 10 μg/ml Leupeptin, and 100 μM Sodium Vanadate, and 20 μg/ml TLCK using a polytron (Brinkmann Instruments, Westbury, NY). The resultant supernatants after centrifugation (14,000 × g for 20 min at 4 °C) were analyzed by Western blot analysis immediately or stored at −80 °C until used.

### Total Lung collagen Accumulation for Fibrosis Analysis

The whole lung collagen level was determined by whole lung hydroxyproline level. The harvested lungs were hydrolyzed in 6 M HCl at 110 °C for 24 hours, and the amount of hydroxyproline in the above lung acid-hydrolysates was performed by colorimetric assay as described previously^58^.

### Rac Activation Assays for Cell Migration Signaling

It is well known that Rac activation leads to increased cell migration. Rac activation was determined by the level of GTP-bound form of Rac in lung tissues *in vivo*. Lung tissues were harvested from control and experimental animal groups and whole lung lysates were prepared as described above. Same amount of cell or tissue lysates were reacted with agarose-bound p21-activated kinase-1 binding domain and co-immunoprecipitates were analyzed by SDS-PAGE gel followed by Western blotted and anti-Rac IgG. Total Rac protein levels were examined by Western blots and the total Rac protein levels were used to analyze the ratio of Rac activation and also serve as a loading control.

### Statistics

Student t-test was used to analyze and compare the data between two groups (Sigma Plot). Data all were pooled and shown as means ± SE. Results were pooled from three to four experiments. For animal studies, each experimental or control group contained 5 to 6 animals and repeated twice. A *p* value of < 0.05 was considered statistically significant.

## Additional Information

**How to cite this article:** Lu, Y.-Y. *et al*. Interaction of Src and Alpha-V Integrin Regulates Fibroblast Migration and Modulates Lung Fibrosis in A Preclinical Model of Lung Fibrosis. *Sci. Rep.* **7**, 46357; doi: 10.1038/srep46357 (2017).

**Publisher's note:** Springer Nature remains neutral with regard to jurisdictional claims in published maps and institutional affiliations.
